# Subtypes of Enteric Neurons in Hirschsprung Disease: A Comparison of Fully Ganglionic and Transitional Zone

**DOI:** 10.1111/nmo.70444

**Published:** 2026-09-25

**Authors:** C. Nebeling, M. Lee‐Theilen, E. Gradhand, F. Friedmacher, U. Rolle, Y. Braun

**Affiliations:** ^1^ Department of Pediatric Surgery and Pediatric Urology Goethe‐University Frankfurt, University Hospital Frankfurt am Main Germany; ^2^ Goethe University Frankfurt, University Hospital, Dr. Senckenberg Institute of Pathology Frankfurt am Main Germany

**Keywords:** enteric nervous system, Hirschsprung disease, neuronal subtypes, transitional zone

## Abstract

**Background:**

Hirschsprung disease (HD) is characterized by aganglionosis of the distal bowel and an adjacent transitional zone with structural abnormalities of the enteric nervous system (ENS). While previous studies have focused primarily on neuronal density, it remains unclear whether the neurochemical composition of enteric neurons differs between transitional and fully ganglionic bowel. This study therefore aimed to characterize neuronal subtype composition in these regions of HD specimens.

**Methods:**

Colonic specimens resected during pullthrough surgery from pediatric HD patients were analyzed. Chromogenic immunohistochemistry and multiplex fluorescent immunohistochemistry (HuC/D, nNOS, ChAT) were performed and analyzed across myenteric and submucosal plexuses. Digital image analysis (QuPath) enabled automated cell detection and subtype classification. Cell counts were normalized per segment length for density analysis. Neuronal subtype composition was analyzed by paired testing and complementary Bayesian Dirichlet–multinomial regression modeling.

**Key Results:**

HuC/D‐, nNOS‐, and ChAT‐positive neurons were identified in both transitional and fully ganglionic bowel, with only minor differences in neuronal density. Paired analysis and Bayesian Dirichlet–multinomial modeling of neuronal subtype proportions indicated that the relative composition of major neurochemical neuronal subtypes was largely preserved between transitional and fully ganglionic bowel. Although expected differences in subtype distribution were observed between enteric plexuses, no consistent zone‐specific alterations were identified.

**Conclusions & Inferences:**

Neurochemical subtype composition of enteric neurons is largely preserved between transitional and fully ganglionic bowel in HD. Within the limitations of this study, we did not detect major differences in neuronal subtype composition between transitional and fully ganglionic bowel that could explain persistent postoperative bowel dysfunction.

## Key Points and Highlights

- Enteric nerve cell subtypes were largely similar in the transitional and fully ganglionic bowel of children with Hirschsprung disease.
- Differences in nerve cell subtype composition were mainly related to the type of enteric nerve plexus rather than the bowel zone.
- Within the limitations of this study, we found no evidence that major changes in nerve cell subtype composition explain persistent bowel problems after surgery.

## Plain Language Summary

Hirschsprung disease is a congenital condition in which nerve cells are absent from the end of the intestine, causing severe bowel dysfunction. Although surgery removes the affected bowel, many patients continue to experience long‐term bowel problems. We investigated whether the types of nerve cells differ between the transitional zone and the normally ganglionic bowel that remains after surgery. Using immunofluorescence imaging and automated digital analysis, we found that the major nerve cell subtypes were distributed similarly in both regions. This study did not find evidence that persistent postoperative symptoms result from major changes in neuronal subtype composition, leaving open alternative explanations, including more subtle structural or functional abnormalities of the remaining bowel.

## Graphical abstract

Neuronal subtype composition is comparable between transitional and fully ganglionic zones in children with Hirschsprung disease. Thus, our study did not support a major contribution of shifts in neuronal subtype composition to persistent postoperative bowel dysfunction.

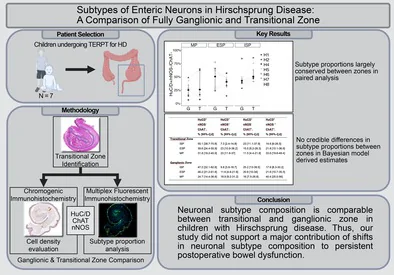

## 1 Introduction

The enteric nervous system (ENS) is a complex neural network embedded within the gastrointestinal tract that autonomously regulates essential functions including motility, secretion, absorption, and local blood flow [1, 2, 3, 4, 5]. Through the coordinated activity of sensory neurons, interneurons, and motor neurons, the ENS integrates luminal, mechanical, and neurohumoral signals to generate reflex circuits that control intestinal function [6, 7, 8, 9]. These processes are further modulated by bidirectional communication with the central nervous system [9, 10, 11].

Within the intestinal wall, enteric neurons are organized into anatomically and functionally distinct plexuses [12]. The myenteric plexus (MP), located between the circular and longitudinal muscle layers, primarily regulates gastrointestinal motility by coordinating smooth muscle contraction and relaxation [13, 14]. In contrast, the external submucosal plexus (ESP), adjacent to the tela muscularis, and the internal submucosal plexus (ISP), located near the lamina muscularis mucosae, are predominantly involved in the regulation of secretion, epithelial barrier function, and local blood flow [15, 16, 17].

Enteric neurons comprise a multitude of diverse subtypes that can be distinguished based on their neurochemical profiles [8, 18, 19]. In particular, the expression of neuronal nitric oxide synthase (nNOS) and choline acetyltransferase (ChAT) allows classification into inhibitory (nitrergic) and excitatory (cholinergic) neuronal populations, respectively [20, 21, 22]. Immunofluorescence‐based approaches have enabled the identification and quantitative assessment of these neuronal subpopulations, demonstrating region‐specific variation in their relative distribution under physiological and pathological conditions [23, 24, 25].

Hirschsprung disease (HD) is a congenital enteric neuropathy characterized by the absence of ganglion cells in variable lengths of the distal intestine, resulting in severe dysmotility and functional obstruction [26, 27, 28]. Clinically, this manifests as delayed meconium passage and chronic constipation, and definitive treatment requires surgical resection of the affected bowel segment [29, 30, 31]. Accurate determination of the proximal resection margin is critical for postoperative outcome but remains challenging due to a transitional zone, which exhibits both quantitative and qualitative abnormalities of the ENS [32, 33]. Previous studies of the transitional zone and fully ganglionic bowel in HD have predominantly focused on neuronal density, whereas the neurochemical composition and subtype distribution of enteric neurons and their importance for postoperative motility disorders remain insufficiently investigated [34, 35].

In this study, we aimed to characterize the neurochemical coding of enteric neurons in HD, with particular emphasis on their abundance and relative subtype distribution across ENS plexuses and between transitional and fully ganglionic bowel segments. For this purpose, we analyzed the full extent of the transitional zone and the adjacent fully ganglionic bowel segment using chromogenic immunohistochemistry and multiplex fluorescent immunohistochemistry approaches combined with statistical modeling to resolve neuronal subtype composition across anatomical and functional compartments.

## 2 Materials and Methods

### 2.1 Patient Samples

Samples were acquired from seven patients (5 male, 2 female) with histologically confirmed HD during pullthrough surgery. Age at the time of surgery ranged from 3 to 54 months (median age 12 months); clinical data are summarized in Table 1.

**TABLE 1:** List of included HD patients.

ID	Sex	Age in months	Associated syndrome	Length of resected bowel in cm	Postoperative symptoms
H1	♂	4		15	HAEC
H2	♂	4		11.5	/
H4	♀	54		28	Constipation
H5	♀	12	Down syndrome	31	Constipation
H6	♂	3		7	/
H7	♂	22	Mowat–Wilson syndrome	18	/
H8	♂	28	Down syndrome	20	Constipation/Soiling

Each resected colon specimen was transferred to the pathology unit. First, tissue specimens for routine histopathological examination, which were not included in the study, were collected. These consisted of 0.5 cm segments from the oral and aboral resection margins, as well as a “Swiss roll” preparation of the remaining bowel, as previously described [36]. Subsequently, specimens for the present study were obtained. The macroscopic transitional zone, defined by the abrupt change in bowel diameter, was identified. From this region, up to three consecutive tissue strips, each measuring a maximum of 5 cm in length and 0.5 cm in width, were excised from the antimesenteric border to encompass the macroscopic transition zone together with sufficient adjacent tissue on the oral side. The oral end of each specimen was marked with tissue dye to preserve orientation throughout histological processing. These samples were then fixed in 4% PBS‐buffered formaldehyde and embedded in paraffin. Three‐μm‐thick cuts were made for hematoxylin and eosin (H&E) staining and chromogenic immunohistochemistry staining, and 10‐μm‐thick cuts for fluorescent immunohistochemistry staining using a microtome and mounted on SuperFrost‐Plus slides (Thermo Scientific, Braunschweig, Germany).

### 2.2 H&E Staining

Formalin‐fixed, paraffin‐embedded (FFPE) tissue sections were deparaffinized and hydrated. Afterward, slides were stained in Mayer's hematoxylin for 4 min, rinsed in tap water, and washed with deionized water 3 times. The slides were then counterstained with alcoholic eosin for 1 min. Afterwards, they were dehydrated in ethanol and mounted in xylene. H&E stainings were used for the definition of ganglionic and transitional zones according to Kapur et al. [33].

### 2.3 Chromogenic Immunohistochemistry Staining

Chromogenic immunohistochemistry staining was performed semi‐automatically using the Leica Bond III system (Leica Biosystems, Nußloch, Germany). Briefly, FFPE tissue sections were placed in a 37°C incubator overnight, deparaffinized, and rehydrated. Heat‐induced epitope retrieval was conducted. Endogenous peroxidase activity was subsequently blocked to minimize nonspecific background staining. Sections were then incubated with the primary antibody at the appropriate dilution, followed by a horseradish peroxidase–conjugated secondary antibody. Primary antibodies are specified in Table 2. Immunoreactivity was visualized using 3,3′‐diaminobenzidine (DAB) as a chromogen, resulting in a brown precipitate at the site of antigen localization. Finally, sections were counterstained with hematoxylin and coverslipped for light microscopic evaluation.

**TABLE 2:** List of antibodies.

Name	Host	Lot number	Dilution	Company
Primary antibodies				
Chromogenic				
Immunohistochemistry				
HuC/D	Rabbit	EPR19098	1:100	Abcam, Cambridge, USA
nNOS	Rabbit	160,870	1:200	Cayman Chemical, Ann Arbor, USA
ChAT	Rabbit	EPR13024 (B)	1:100	Abcam, Cambridge, USA
p75 (NGF)	Rabbit	EP1039 (Y)	1:200	Abcam, Cambridge, USA
VIP	Rabbit	EPR23288‐43	1:100	Abcam, Cambridge, USA
Primary antibodies				
Fluorescent				
Immunohistochemistry				
HuC/D	Mouse	16A11	1:50	Thermo Scientific, Braunschweig, Germany
nNOS	Rabbit	160,870	1:200	Cayman Chemical, Ann Arbor, USA
ChAT	Goat	NBP1‐30052	1:100	Novus Biologicals, Centennial, USA
DAPI	—	P36931	—	Thermo Scientific, Braunschweig, Germany
Secondary antibodies				
Anti‐Goat CF555	Donkey	20,039–1	1:500	Biotium, Fremont, USA
Anti‐Rabbit CF488	Donkey	20,015–1	1:500	Biotium, Fremont, USA
Anti‐Mouse CF633	Donkey	20,124–1	1:500	Biotium, Fremont, USA

### 2.4 Multiplex Fluorescent Immunohistochemistry Staining

For fluorescent immunohistochemistry staining, FFPE tissue sections were deparaffinized and rehydrated, followed by two 5‐min washes in deionized water. A hydrophobic barrier was drawn around the tissue to confine reagents and ensure uniform distribution. Heat‐induced epitope retrieval was performed in 1 × R‐Universal buffer (Aptum Biologicals, Southampton, Great Britain) at sub‐boiling temperature for 10 min, followed by a 30‐min cooling period and two washes in PBS. Sections were then incubated in PBS containing 1% donkey serum and 0.4% Triton X‐100 (2 × 10 min) and subsequently blocked with 5% donkey serum for 1 h at room temperature. Primary antibody incubation was carried out with 200 μL antibody solution diluted in 1% donkey serum for 1 h at room temperature. After washing in PBS (2 × 10 min), sections were incubated with the corresponding secondary antibody (200 μL, diluted in 1% BSA) for 1 h in a humidified chamber. Primary and secondary antibodies are listed in Table 2. Following additional PBS washes (2 × 10 min), slides were mounted using ProLong Gold Antifade Reagent with DAPI (Thermo Scientific, Braunschweig, Germany) and coverslipped. Samples were allowed to cure at 4°C for 24 h prior to fluorescence microscopic analysis.

### 2.5 Image Acquisition

H&E‐stained as well as IHC‐stained slides were scanned using a Pannoramic Scan II (3DHistech, Budapest, Hungary). Fluorescent immunohistochemistry slides were digitized using a Carl Zeiss Axio Scan 7 (Carl Zeiss, Jena, Germany) in the “IF” mode. All resulting whole‐slide images (WSIs) were imported into open‐source software (QuPath v0.5.1) for viewing and further assessment [37].

### 2.6 Digital Image Analysis

#### 2.6.1 Annotation

Regions of interest (ROIs) were manually annotated prior to analysis. Both the submucosal and the myenteric plexuses were delineated on each section. Based on established histopathological criteria, tissue areas were classified as transitional zone or fully ganglionic (i.e., normally innervated) [33]. The lengths of the total specimen and zones were recorded.

#### 2.6.2 Cell Detection

Cell detection was performed using the “Cell detection” module in QuPath. Nuclei were identified automatically based on hematoxylin staining in chromogenic immunohistochemical sections and DAPI fluorescence in fluorescent immunohistochemistry sections. Detection parameters (e.g., minimum and maximum nuclear size, intensity threshold, cell expansion) were predefined and are summarized in Tables S1 and S2, respectively.

#### 2.6.3 Detection of Positive Cells in Chromogenic Immunohistochemistry

Following ROI annotation, positive cells in chromogenic immunohistochemically stained sections were identified using the “Positive cell detection” module. Prior to analysis, hematoxylin and DAB intensities were normalized to ensure inter‐slide comparability. Nuclei were detected based on hematoxylin staining, and DAB intensity thresholds were applied to determine cells as positive. Parameter settings were adapted to staining characteristics (e.g., signal intensity, subcellular localization, background) and are summarized in Table S3.

#### 2.6.4 Classification of Enteric Neurons in Fluorescent Immunohistochemistry

Quadruple (DAPI, HuC/D, nNOS, and ChAT) fluorescent immunohistochemistry staining was analyzed using multiplex workflows in QuPath. After DAPI‐based nuclear detection, marker‐specific classifiers were generated using the “Create single measurement classifier” module, defining subcellular compartment (nucleus, cytoplasm, whole cell), measurement type (e.g., mean or maximum intensity), and positivity threshold. The positivity threshold was set after subtracting mean background staining and calculating the mean and variance of staining intensity and defined as “staining intensity > mean staining intensity + standard deviation”. Individual classifiers were subsequently combined using the “Create composite classifier” module, enabling sequential execution and assignment of each detected cell to a defined phenotypic class. Exemplary parameter settings employed are summarized in Table S4, and an exemplary classification of ganglion cells is depicted in Figure S1.

#### 2.6.5 Data Export

For each detected cell within annotated ROIs, QuPath generated quantitative metrics including morphometric parameters and the number of positive cells per classifier. Data were exported as CSV files for downstream statistical analysis.

#### 2.6.6 Automated Analysis Scripts

Custom scripts were developed within the QuPath script editor to standardize and automate the described workflows. Separate scripts were established for each chromogenic immunohistochemical staining and for multiplex fluorescent immunohistochemistry‐based cell classification to ensure reproducibility across sections.

### 2.7 Statistical Analysis

Data were organized using Microsoft Excel and analyzed with R [38]. Descriptive statistics were calculated for all variables. Neuronal density was expressed as the number of positive cells per mm of the respective bowel segment. Neuronal subtype composition was expressed as the proportion of each subtype relative to the total number of HuC/D‐positive neurons. The number of HuC/D‐positive neurons analyzed per patient and plexus is depicted in Table S5.

For the statistical analysis of neuronal subtype composition, proportions were calculated separately for each patient, plexus, and zone by dividing the total number of neurons classified into each subtype across all analyzed sections for that patient and compartment by the corresponding number of HuC/D‐positive neurons. Transitional and fully ganglionic zones from the same patient were compared using the paired Wilcoxon signed rank test and the paired *t*‐test as a sensitivity analysis with Holm correction. Bayesian Dirichlet–multinomial regression modeling was used as a complementary analysis of neuronal subtype composition. In brief, the total number of enteric neurons per observation was included as a trials term, and specimen identity was included as a random intercept to account for repeated measures. Fixed effects included layer, zone, and their interaction. Models were fitted using the brms package in R, with 4 chains of 4000 iterations each and an adapt_delta of 0.99 to ensure stable sampling [39].

## 3 Results

### 3.1 Single Expression of Neuronal Markers in Enteric Neurons

**FIGURE 1:**
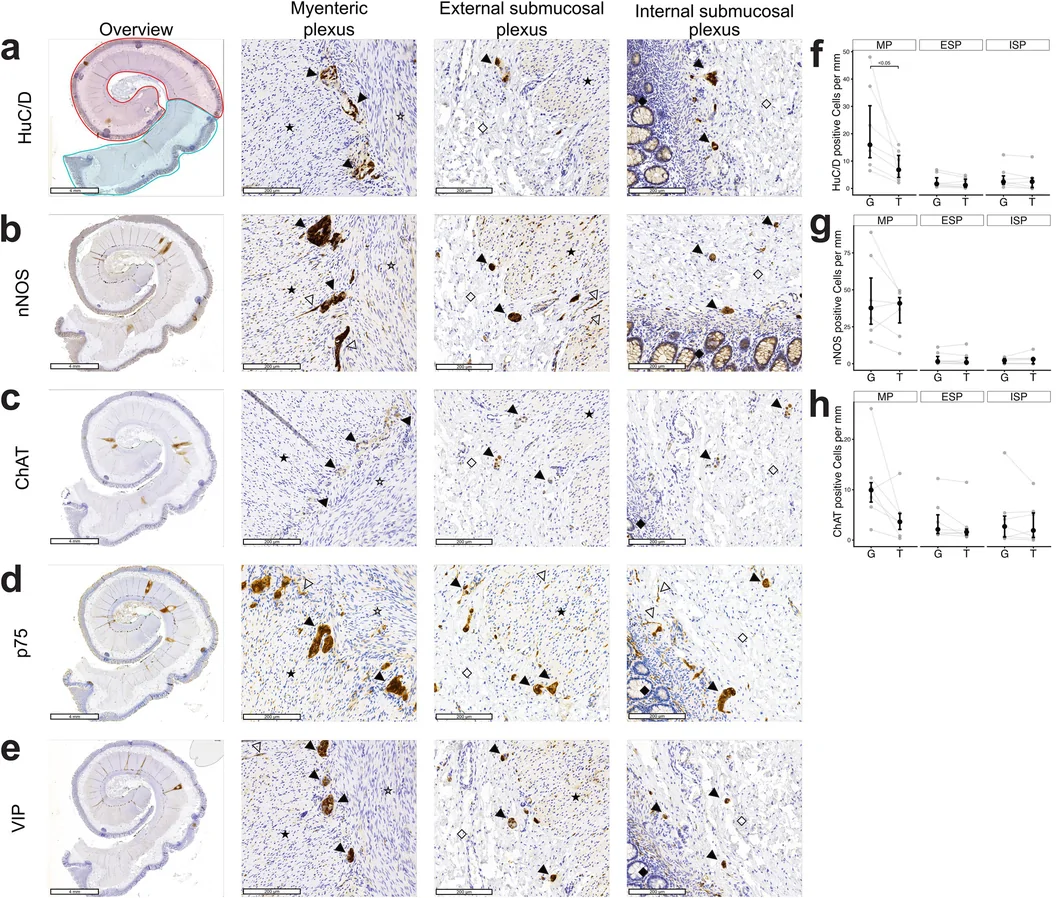
Overview and higher magnifications (ganglionic zone) of both submucosal and the myenteric plexus for each staining: HuC/D (a), nNOS (b), ChAT (c), p75 (d), VIP (e). The extent of the transitional (light blue) and ganglionic zone (light red) are depicted with color overlay in the upper left image. Hollow stars, outer muscle layer; full stars, inner muscle layer; hollow diamond, submucosal layer; full diamond, mucosal layer; hollow arrowheads, positive nerve fibers; full arrowheads, positive ganglia. Spaghetti plots—HuC/D (f), nNOS (g), ChAT (h) depicting the number of positive cells per millimeter in each specimen in ganglionic and transitional zone as connected dots in light gray, overall median and IQR are shown in black. Analysis is split per plexus (MP, myenteric plexus; ESP, external submucosal plexus; ISP, internal submucosal plexus).

We first assessed the expression of the pan‐neuronal marker HuC/D and the major neuronal enzymes nNOS and ChAT in the myenteric and submucosal (external and internal) plexuses across transitional and fully ganglionic segments of the HD specimens using chromogenic immunohistochemistry. HuC/D immunoreactivity was confined to neurons in all plexuses of the ENS, with no detectable staining of glia or nerve fibers (Figure 1a). Quantitative analysis revealed a higher number of HuC/D‐positive cells per millimeter in the fully ganglionic zone compared to the transitional zone segments; however, only the difference in the myenteric plexus reached statistical significance (MP: 15.9 (19) vs. 6.82 (8.05) *p* = 0.0469; ESP: 1.7 (2.7) vs. 1.2 (2.9), *p* = 0.219; ISP: 2.4 (3.0) vs. 2.48 (3.5), *p* = 0.219) (Figure 1f).

Similarly, nNOS‐positive cells were identified in both transitional and fully ganglionic zones (Figure 1b). In contrast to HuC/D and ChAT, nNOS immunoreactivity was additionally observed in hypertrophic nerve fibers, including those within aganglionic regions. Quantitative comparison demonstrated no significant difference in nNOS‐positive cell density between transitional and fully ganglionic segments (MP: 37.7 (31.3) vs. 40.9 (17.2), *p* = 0.891; ESP: 1.5 (4.3) vs. 0.9 (3.7), *p* = 0.891; ISP: 2.2 (3.1) vs. 3.0 (3.5), *p* = 0.891) (Figure 1g).

ChAT expression was restricted to enteric neurons and was detected in all ENS plexuses of transitional and fully ganglionic zones (Figure 1c). Comparable to HuC/D, the mean number of ChAT‐positive cells per millimeter was slightly higher in fully ganglionic segments, though this difference was not statistically significant (MP: 9.9 (3.9) vs. 3.6 (3.2) *p* = 0.234; ESP: 2.1 (3.7) vs. 1.6 (1.2), *p* = 0.234; ISP: 2.7 (4.1) vs. 1.9 (4.9), *p* = 0.578) (Figure 1h). Full statistical analysis is summarized in Table S6. Because of the age heterogeneity in our study cohort, we additionally performed a subgroup analysis of samples collected during surgery before the end of the first year of life. The observed trends were comparable to those in the full cohort (Table S7).

Next, the expression of vasoactive intestinal peptide (VIP) and the neurotrophin receptor p75 were examined. Both markers were detected throughout the bowel—including aganglionic, transitional, and fully ganglionic segments—in all ENS components, including enteric neurons, nerve fibers, and glial cells (Figure 1d,e). Due to their ubiquitous distribution across neurons, glial cells, and nerve fibers, quantitative analysis was not performed.

### 3.2 Multiplex Fluorescent Immunohistochemistry Reveals Enteric Neuron Subtypes

**FIGURE 2:**
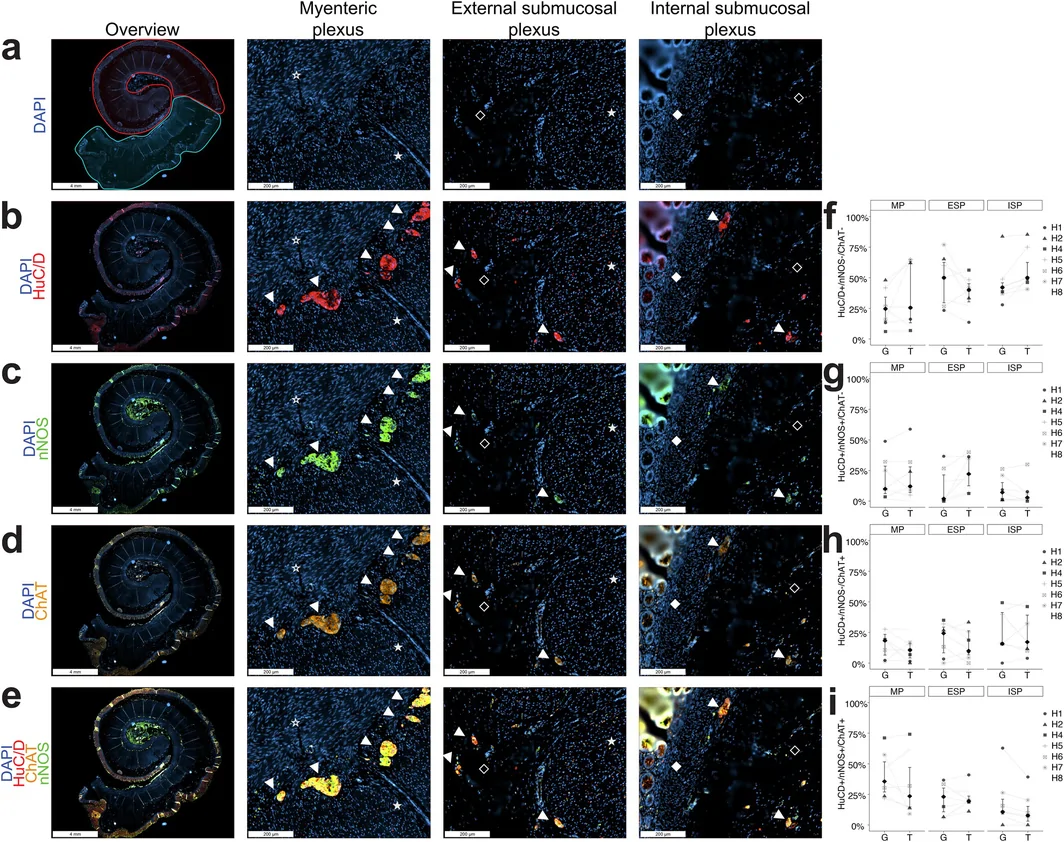
Overview and higher magnifications (ganglionic zone) of both submucosal and the myenteric plexus for different combinations of staining: DAPI (a), DAPI/HuC/D (b), DAPI/nNOS (c), DAPI/ChAT (d), DAPI/HuC/D/nNOS/ChAT (e). The extent of the transitional (light blue) and ganglionic zone (light red) are depicted with color overlay in the upper left image. Hollow stars, outer muscle layer; full stars, inner muscle layer; hollow diamond, submucosal layer; full diamond, mucosal layer; hollow arrowheads, positive nerve fibers; full arrowheads, positive ganglia. Spaghetti plots—HuC/D^+^/nNOS^−^/ChAT^−^ (f), HuC/D^+^/nNOS^+^/ChAT^−^ (g), HuC/D^+^/nNOS^−^/ChAT^+^ (h), HuC/D^+^/nNOS^+^/ChAT^+^ (i) depicting the proportion of each subtype in each specimen across ganglionic and transitional zone. Patient specific proportions are shown with connected individual symbols. Overall median and IQR are shown in black. Analysis is split by plexus (MP, myenteric plexus; ESP, external submucosal plexus; ISP, internal submucosal plexus).

Single‐marker analyses do not allow reliable neuronal subtype discrimination; therefore, we performed multiplex fluorescent immunohistochemistry using DAPI for nuclear identification, HuC/D as a pan‐neuronal marker, and nNOS and ChAT to define nitrergic and cholinergic subpopulations, respectively (Figure 2a–e).

Consistent with the previous immunohistochemical analysis, HuC/D^+^/nNOS^−^/ChAT^−^ neurons were detected in all plexuses of both transitional and fully ganglionic segments and the cell density was slightly higher in fully ganglionic segments, although this difference did not achieve statistical significance (MP: 2.6 (4.0) vs. 0.6 (2.1), *p* = 0.094; ESP: 1.4 (1.7) vs. 0.6 (0.6), *p* = 0.094; ISP: 2.1 (2.0) vs. 1.2 (0.8), *p* = 0.094) (Figure S2a).

Subtype analysis demonstrated that both HuC/D^+^/nNOS^+^/ChAT^−^ and HuC/D^+^/nNOS^−^/ChAT^+^ neurons were present in all plexuses and in both bowel segments. The density of nitrergic (MP: 2.6 (5.7) vs. 0.7 (4.2) *p* = 0.75; ESP: 0.2 (0.9) vs. 0.4 (0.8), *p* = 1; ISP: 0.1 (0.7) vs. 0.06 (0.2), *p* = 0.141) and cholinergic (MP: 2.4 (2.0) vs. 0.4 (0.9) *p* = 0.234; ESP: 0.7 (0.9) vs. 0.3 (0.4), *p* = 0.313; ISP: 0.8 (0.7) vs. 0.3 (0.2), *p* = 0.313) neurons did not differ significantly between transitional and fully ganglionic zones (Figure S2b,c).

Triple‐positive HuC/D^+^/nNOS^+^/ChAT^+^ neurons were predominantly observed in the myenteric plexus of fully ganglionic segments and were only rarely detected in other ENS compartments (Figure 2e). Although numerically enriched in the myenteric plexus, their density did not differ significantly between fully ganglionic and transitional zones (MP: 7.0 (4.0) vs. 2.7 (3.6), *p* = 0.188; ESP: 0.5 (0.9) vs. 0.7 (0.5), *p* = 1; ISP: 0.5 (0.6) vs. 0.1 (0.4), *p* = 0.211) (Figure S2d).

Full statistical analysis is summarized in Table S8. Again, we additionally performed a subgroup analysis of all samples collected during surgery before completion of the first year of life and observed similar trends in comparison to the full cohort (Table S9).

### 3.3 Distribution of Neuronal Subtypes Across Zones and Plexuses

To further characterize the overall ENS composition in the analyzed colonic specimen, we pooled the absolute number of neurons classified in each subtype across all patients for each plexus and zone. The relative proportion of each neuronal subtype was then calculated from the total number of HuC/D‐positive neurons within the respective plexus and zone. The majority of analyzed enteric neurons were HuC/D^+^/nNOS^−^/ChAT^−^ positive. HuC/D^+^/nNOS^+^/ChAT^−^ and HuC/D^+^/nNOS^+^/ChAT^+^ neurons each accounted for approximately 19%–25% of neurons, whereas HuC/D^+^/nNOS^−^/ChAT^+^ neurons represented the smallest proportion in both zones (Table 3). Overall, the pooled relative distribution of neuronal subtypes was comparable between transitional and fully ganglionic bowel.

**TABLE 3:** Percentage of neuronal subtypes across zones and layers.

	HuC/D^+^ nNOS^−^ ChAT^−^, (%)	HuC/D^+^ nNOS^+^ ChAT^−^, (%)	HuC/D^+^ nNOS^−^ ChAT^+^, (%)	HuC/D^+^ nNOS^+^ ChAT^+^, (%)
Transitional zone	**40.1**	**21.8**	**12.3**	**25.8**
ISP	51	21	17.8	10.2
ESP	41.6	20.3	20.3	17.7
MP	35.2	22.8	6.4	35.6
Ganglionic zone	**45.8**	**19.5**	**15.5**	**19.2**
ISP	51.9	15.3	23.6	9.2
ESP	52.4	16.9	22.3	8.5
MP	41.5	22.4	11.6	24.5

*Note:* Values represent the pooled percentage of each neuronal subtype within the indicated plexus and zone, calculated from the summed neuron counts across all patients. Bold values indicate overall distribution across all plexuses in both zones.

Abbreviations: ESP, external submucosal plexu; ISP, internal submucosal plexus; MP, myenteric plexus.

A higher proportion of HuC/D^+^/nNOS^−^/ChAT^−^ neurons was seen in both submucosal plexuses, while triple‐positive neurons were consistently the rarest subtype in these compartments across both zones. In fully ganglionic submucosal plexuses, HuC/D^+^/nNOS^−^/ChAT^+^ neurons were relatively more abundant than HuC/D^+^/nNOS^+^/ChAT^−^ neurons, whereas in the submucosal plexuses in the transitional zone, both double‐positive populations showed similar frequencies.

Additionally, we performed paired comparison analysis of the patient‐level proportions of each neuronal subtype between the transitional and fully ganglionic zones within each plexus. In contrast to the pooled proportions presented in Table 3, these analyses were performed separately for each patient, with patient identity representing the biological replicate. The largest median difference was observed for HuC/D^+^/nNOS^−^/ChAT^−^ neurons, with a higher proportion in the transitional compared with the fully ganglionic zone of the internal submucosal plexus, although this difference did not reach statistical significance (*p* = 0.068) (Table 4). Differences in proportions of the other neuronal subtypes were less pronounced, and no statistically significant differences were identified. Individual patient‐level proportions underlying these comparisons are shown in Figure 2f–i, and the complete statistical analysis is provided in Table S10.

**TABLE 4:** Paired comparison of relative proportions of neuronal subtypes between both zones across all plexuses.

Plexus	Ganglionic median, % (IQR)	Transitional median, % (IQR)	Median difference	Adjusted *p*
HuC/D^+^/nNOS^−^/ChAT^−^				
ISP	42.1 (8.1)	50 (14.9)	7.5	0.068
ESP	50 (33.1)	40 (14.8)	9.7	1
MP	24.6 (19.4)	25.5 (54.8)	0.8	1
HuC/D^+^/nNOS^+^/ChAT^**−**^				
ISP	7.1 (14.1)	2.9 (0.6)	1.3	1
ESP	2 (20.7)	22.2 (23)	4.9	0.178
MP	10 (22.5)	12.1 (21)	0.4	1
HuC/D^+^/nNOS^−^/ChAT^+^				
ISP	15.8 (25.7)	17.2 (28.3)	3.2	1
ESP	24.3 (21.1)	9.1 (24.1)	2.9	1
MP	18.4 (17)	10.6 (12.6)	2.2	0.615
HuC/D^+^/nNOS^+^/ChAT^+^				
ISP	10.7 (12.2)	7.7 (12)	5.8	0.108
ESP	23.1 (19.4)	19.5 (8.7)	0.2	1
MP	35.6 (24.7)	23.5 (32.6)	6.7	0.894

*Note:* Values represent the median percentage (IQR) of each neuronal subtype across patients for the respective zone and plexus. *p*‐values were derived from paired patient‐level comparisons between transitional and fully ganglionic zones using the Wilcoxon signed‐rank test.

Abbreviations: ESP, external submucosal plexus; ISP, internal submucosal plexus; MP, myenteric plexus.

The largest median difference was observed for HuC/D^+^/nNOS^−^/ChAT^−^ neurons, with a higher proportion in the transitional compared with the fully ganglionic zone of the internal submucosal plexus, although this difference did not reach statistical significance (*p* = 0.068) (Table 4). Differences in the proportions of the other neuronal subtypes were less pronounced, and no statistically significant differences were identified.

### 3.4 Bayesian Modeling of Neuronal Subtype Composition

To complement our paired patient‐based analyses of neuronal subtype composition, a Bayesian Dirichlet–multinomial model was used to evaluate neuronal subtype composition jointly while accounting for the compositional structure of the data, differences in the number of neurons quantified between specimens, overdispersion, and specimen‐level heterogeneity. As expected, substantial differences in neuronal subtype composition were observed between the myenteric and submucosal plexuses. In contrast, zone effects and plexus‐by‐zone interactions showed substantial uncertainty, with credible intervals overlapping zero for all comparisons. Model‐derived posterior proportions are shown in Table 5. Overall, the model provided no evidence for a major shift in neuronal subtype composition between transitional and fully ganglionic bowel (Table S11).

**TABLE 5:** Bayesian estimation of distribution of neuronal subtypes across layers and zones.

	HuCD^+^ nNOS^−^ ChAT^−^, % [95%‐CrI]	HuCD^+^ nNOS^+^ ChAT^−^, % [95%‐CrI]	HuCD^+^ nNOS^−^ ChAT^+^, % [95%‐CrI]	HuCD^+^ nNOS^+^ ChAT^+^, % [95%‐CrI]
Transitional zone				
ISP	55.1 [38.7–70.8]	7.3 [2.4–14.9]	23 [11.1–37.9]	14.6 [6–26.5]
ESP	39.6 [24.4–55.9]	23 [10.9–38.2]	15.5 [6.2–28.3]	21.8 [10.1–36.9]
MP	31.8 [19.2–45.9]	23 [11.6–37]	11.5 [4.4–21.9]	33.6 [19.6–49.4]
Ganglionic zone				
ISP	47.2 [32.1–62.8]	9.8 [3.8–18.7]	25.2 [13–39.5]	17.8 [8.3–30.2]
ESP	46.2 [31.2–61.6]	11.6 [4.6–21.6]	20.6 [10–34]	21.6 [10.7–35.3]
MP	24.7 [14.4–36.8]	18.9 [9.2–31.2]	16 [7.5–26.8]	40.4 [25.5–56]

*Note:* Values represent model‐derived posterior mean estimates of the proportion of each neuronal subtype for the indicated plexus and zone, with 95% credible intervals.

Abbreviations: ESP, external submucosal plexus; ISP, internal submucosal plexus; MP, myenteric plexus.

## 4 Discussion

A detailed characterization of the ENS abnormalities in HD beyond the absence of ganglion cells in the distal intestine is essential to understand disease etiology, genotype–phenotype correlation, and persistent gastrointestinal symptoms experienced by many patients following corrective surgery. Accordingly, this study investigated the neurochemical composition of enteric neurons throughout the transitional and fully ganglionic bowel across all major enteric plexuses. Previous investigations predominantly focused on morphological alterations and neuronal density to define the transitional zone and guide surgical resection [33, 34, 35]. More recently, single‐cell RNA‐sequencing and spatial transcriptomics have demonstrated that the ENS comprises a remarkable diversity of molecularly and functionally distinct neuronal populations rather than only a few neurochemical classes [8, 40, 41, 42, 43]. Together with advances in tissue preparation and 3D imaging, these studies have considerably expanded our understanding of ENS organization [44, 45]. Consequently, postoperative dysfunction in HD may result from subtle alterations in neuronal composition, connectivity, or function that extend beyond conventional histopathological assessment [46, 47].

To investigate the major functional neuronal subpopulations of HD patients, we employed single‐marker chromogenic immunohistochemistry together with multiplex fluorescent immunohistochemistry using HuC/D, nNOS, and ChAT. Quantitative analyses demonstrated largely preserved neuronal densities and subtype distributions between the transitional and fully ganglionic bowel. Although fully ganglionic segments generally exhibited slightly higher neuronal densities than the transitional zone, statistically significant differences were limited to HuC/D‐positive neurons within the myenteric plexus.

The largest neuronal population in most plexuses consisted of HuC/D^+^/nNOS^−^/ChAT^−^ neurons. This population should not be interpreted as representing a single neuronal subtype. Instead, because our analysis was intentionally restricted to the two principal functional markers nNOS and ChAT, this category likely comprises a heterogeneous mixture of enteric neurons, including interneurons, intrinsic primary afferent neurons, and excitatory or inhibitory motor neurons expressing alternative neurochemical markers such as calretinin, calbindin, substance P, somatostatin, neuropeptide Y, or CGRP [18]. Consequently, our classification represents a simplified neurochemical framework that captures the major nitrergic and cholinergic populations but does not encompass the full molecular diversity of the ENS now recognized by transcriptomic studies. Nevertheless, this targeted approach enables robust quantitative comparison between bowel regions using markers that are well established.

The relatively large proportion of HuC/D^+^/nNOS^−^/ChAT^−^ neurons compared with previous reports may also reflect methodological differences [25, 48, 49]. Digital pathology with predefined positivity thresholds minimizes observer bias and enables reproducible high‐throughput analysis but may classify neurons expressing only low levels of nNOS or ChAT as marker‐negative. In contrast, manual observer‐based assessment may identify weakly stained cells as positive, resulting in lower proportions of apparently marker‐negative neurons. Therefore, direct comparison of absolute subtype proportions across studies should be interpreted cautiously, particularly considering differences in antibodies, staining protocols, image analysis strategies, tissue preparation, and patient age [50].

Consistent with previous reports, nNOS‐positive neurons were present throughout all plexuses, whereas nNOS immunoreactivity was additionally detected within hypertrophic nerve fibers, particularly in the transitional and aganglionic bowel. Multiplex immunofluorescence demonstrated substantially fewer HuC/D^+^/nNOS^+^/ChAT^−^ neurons than nNOS‐positive cells observed by chromogenic immunohistochemistry, indicating that a considerable fraction of nNOS immunoreactivity originates from neuronal processes and adjacent cells rather than neuronal cell bodies. Importantly, neither absolute neuronal density nor relative proportions of nitrergic neurons differed substantially between transitional and fully ganglionic bowel. Previous studies have associated increased nitrergic signaling with constipation and Hirschsprung‐associated enterocolitis [35, 51, 52]; however, our present study did not support these associations.

Similarly, cholinergic neurons were distributed throughout all plexuses, with slightly higher densities observed in fully ganglionic bowel. Although cholinergic hyperinnervation has been described within aganglionic nerve fibers and reduced ChAT expression has been reported in ganglionic bowel of HD patients compared with controls [35, 53, 54], our analyses did not reveal substantial compositional differences between the transitional and fully ganglionic zones. These findings did not support the notion that alterations in cholinergic neuronal subtype composition represent a dominant mechanism underlying postoperative bowel dysfunction.

Enteric neurons are often described as exclusively nitrergic or cholinergic [55, 56]. However, almost all neurochemical investigations describe a subpopulation of ChAT^+^/nNOS^+^ neurons to a varying abundance [49, 54, 56]. If this is a veritable distinct population or the detection of these cells represents technical shortcomings is yet to be determined, also in emerging single‐cell‐transcriptomic data [57]. We also observed a substantial amount of HuC/D^+^/ChAT^+^/nNOS^+^ cells in our samples, especially in the myenteric plexus. Overlap between cell bodies in microscopic imaging may contribute to incorrect detection of positive expression, especially in ganglia with high neuronal cell density. Nonetheless, with consistent methodology, the comparison between zones and plexuses remains feasible in a specific cohort. However, we could not observe significant differences in HuC/D^+^/ChAT^+^/nNOS^+^ cells per mm between zones and plexuses.

Initially, we also evaluated VIP and p75 because both markers have previously been proposed to further characterize enteric neuronal subpopulations [49, 58]. However, both proteins exhibited widespread immunoreactivity throughout neuronal cell bodies, nerve fibers, and glial elements across ganglionic, transitional, and aganglionic bowel. This diffuse staining precluded reliable assignment of VIP or p75 expression to individual HuC/D‐positive neurons within our multiplex analysis workflow. Therefore, we focused on nNOS and ChAT, which provided robust neuronal subtype classification suitable for automated quantitative analysis.

Beyond absolute neuronal densities, we analyzed neuronal subtype composition using proportional analyses and Bayesian Dirichlet–multinomial modeling to account for unequal neuronal counts and the compositional nature of the data. Both approaches consistently demonstrated that differences between bowel wall layers were more pronounced than differences between transitional and fully ganglionic bowel. The myenteric plexus was characterized by relatively higher proportions of nitrergic and triple‐positive neurons, whereas both submucosal plexuses contained proportionally fewer inhibitory neuronal populations. These findings are consistent with the established functional specialization of the myenteric plexus in regulating intestinal motility [22]. In contrast, zone‐dependent differences were generally modest and associated with considerable uncertainty, suggesting that neuronal subtype composition remains largely preserved despite the presence of the transitional zone.

Compared with previously published datasets from healthy human colon, our Bayesian model‐derived neuronal subtype proportions show both similarities and notable differences. Consistent with previous reports, nitrergic neurons represented a major neuronal population of the myenteric plexus. However, the estimated proportion of HuC/D^+^/nNOS^+^/ChAT^−^ neurons in our study was lower than reported by Tian et al., who observed a median proportion of 43.3% (IQR 40.0%–44.0%) in the myenteric plexus of healthy controls, and by Chen et al., who reported 41.7% across the colon, compared with 18.9% (95% CrI 9.2%–31.2%) in our Bayesian model [25, 49]. Likewise, the proportion of HuC/D^+^/nNOS^−^/ChAT^+^ neurons was lower in our cohort (16.0% [7.5%–26.8%]) than the values reported by Tian et al. (42.1% (40.3%–44.7%)) and Chen et al. (47.7%). Consequently, the proportion of HuC/D^+^/nNOS^−^/ChAT^−^ neurons was substantially higher in our analysis than in these previous studies (24.7% [14.4%–36.8%] vs. 4.8% (3.1%–6.7%) and 1.8%, respectively), while the proportion of HuC/D^+^/nNOS^+^/ChAT^+^ neurons was also higher (40.4% [25.5%–56%] vs. 2.6% (2.0%–4.6%) and 8.8%, respectively).

These apparent differences should, however, be interpreted with caution. Our study employed an automated digital image analysis workflow with predefined positivity thresholds, whereas the published studies used different antibodies, staining protocols, tissue preparation techniques, and manual assessment strategies. Furthermore, the available reference datasets were derived from different patient populations and age groups. Eisenberg et al., using three‐dimensional tissue visualization, demonstrated considerable physiological variability even among healthy individuals, with the proportion of HuC/D^+^/nNOS^+^ neurons ranging from 34.3% to 50.6% in children and from 43.7% to 63.9% in adults, while HD specimens ranged from 27.8% to 63.3% [45]. These observations highlight the substantial biological and methodological variability inherent to neuronal subtype analyses and emphasize that definitive conclusions regarding disease‐specific alterations require studies including age‐matched healthy controls analyzed using identical experimental and analytical pipelines.

Similarly, an important consideration when interpreting our findings is that the fully ganglionic bowel should not necessarily be regarded as physiologically normal. Increasing evidence suggests that HD may involve diffuse developmental abnormalities of the ENS extending beyond the aganglionic segment [43, 45]. Consequently, the ganglionic bowel retained after pull‐through surgery may itself exhibit subtle structural, molecular, or functional abnormalities that contribute to persistent postoperative symptoms. Our study therefore compares two regions within HD bowel rather than comparing diseased tissue with truly healthy intestine. While this internal comparison allowed us to specifically investigate alterations associated with the transition zone, future studies incorporating healthy pediatric control tissue or advanced transcriptomic datasets will be required to determine whether the ganglionic bowel in HD differs from normal colon.

Our study has several additional limitations. First, the cohort remained relatively small and heterogeneous regarding patient age and associated syndromes, both of which may influence neuronal composition [48, 59, 60]. Although subgroup analyses of infants yielded similar trends, larger age‐stratified cohorts will be necessary to fully address developmental differences. Second, standardized postoperative functional follow‐up was not available, preventing correlation of neuronal subtype composition with long‐term clinical outcomes such as constipation, soiling, or Hirschsprung‐associated enterocolitis. Third, neuronal quantification was performed on thin paraffin sections, providing a two‐dimensional representation of a highly complex three‐dimensional neuronal network. Consequently, neuronal counts may be influenced by ganglion orientation, sectioning plane, and regional variation in plexus distribution around the bowel circumference. Emerging approaches including whole‐mount preparations, tissue clearing, and three‐dimensional microscopy are likely to provide more comprehensive characterization of ENS architecture. Finally, although our automated digital pathology workflow minimizes observer bias and provides reproducible quantitative assessment, differences between automated and manual classification strategies may partly explain discrepancies with previous studies.

## 5 Conclusion

Taken together, although expected differences between enteric plexuses were observed, neuronal subtype composition was largely preserved between transitional and fully ganglionic bowel. These results did not support a major contribution of alterations in the relative abundance of nNOS‐ and ChAT‐defined neuronal subtypes to persistent postoperative bowel dysfunction. Instead, future studies integrating high‐dimensional molecular profiling, functional analyses, and three‐dimensional imaging will be required to determine whether more subtle abnormalities in neuronal identity, connectivity, or circuit organization contribute to persistent gastrointestinal dysfunction after surgical treatment.

## Author Contributions

Conceptualization: C. Nebeling, U. Rolle, Y. Braun; methodology: C. Nebeling, M. Lee‐Theilen, Y. Braun; formal analysis and investigation: C. Nebeling, M. Lee‐Theilen, E. Gradhand, Y. Braun; writing – original draft preparation: U. Rolle, Y. Braun; writing – review and editing: all authors; funding acquisition: Y. Braun; resources: E. Gradhand, F. Friedmacher; supervision: U. Rolle, Y. Braun.

## Funding

This study was supported by a grant from the Rudolf‐Geißendörfer‐Stiftung to YB.

## Ethics Statement

The local ethics committee (Ethik‐Kommission des Fachbereichs Medizin der Goethe‐Universität Frankfurt am Main ‐ Geschäfts‐Nr.: iBDF 274/18) approved this study. All procedures were in accordance with the 1964 Helsinki Declaration and its later amendments.

## Conflicts of Interest

The authors declare no conflicts of interest.

## Supporting information


**Table S1:** Setup parameters for cell detection in chromogenic immunohistochemistry.
**Table S2:** Setup parameters for cell detection in fluorescent immunohistochemistry.
**Table S3:** Setup parameters for positive cell detection in chromogenic immunohistochemistry.
**Table S4:** Setup parameters for positive cell detection in fluorescent immunohistoechmistry.
**Table S5:** Sum of analyzed HuC/D‐positive neurons per patient and plexus.
**Table S6:** Comparison between Transitional and Ganglionic Zone Chromogenic Immunohistochemistry.
**Table S7:** Children under 1 year, comparison between Transitional and Ganglionic Zone chromogenic immunohistochemistry.
**Table S8:** Comparison between Transitional and Ganglionic Zone fluorescent immunohistochemistry.
**Table S9:** Children below 1 year, comparison between Transitional and Ganglionic Zone fluorescent immunohistochemistry.
**Table S10:** Comparison of proportions of each neuronal subtype between Transitional and Ganglionic Zone.
**Table S11:** Bayesian modeling estimation parameters.
**Figure S1:** Exemplary detailed depiction of automated neuronal subtype classification in a single ganglion in the myenteric plexus. Stainings shown are (a) DAPI, (b) DAPI/HuC/D, (c) DAPI/nNOS, (d) DAPI/ChAT. A combined image of all markers was used for classification (e); examples of all identified subtypes of HuC/D positive cells and nNOS positive cells negative for HuC/D are depicted.
**Figure S2:** Spaghetti plot depicting the paired comparison of neuronal density (cells per mm) of the neuronal subtypes investigated between ganglionic and transitional zones and within each plexus in all patients: HuC/D+/nNOS−/ChAT− (a), HuC/D+/nNOS+/ChAT− (b), HuC/D+/nNOS−/ChAT+ (c), HuC/D+/nNOS+/ChAT+ (d). Overall median and IQR are shown in black. Analysis is split by plexus (MP, myenteric plexus; ESP, external submucosal plexus; ISP, internal submucosal plexus).

## Data Availability

The data that support the findings of this study are available from the corresponding author upon reasonable request.
