# Supplementary material for: Subtypes of Enteric Neurons in Hirschsprung Disease: A Comparison of Fully Ganglionic and Transitional Zone
Source: Neurogastroenterol Motil. 2026 Sep 25;38(9):e70444. doi: 10.1111/nmo.70444 (PMC13615294; doi:10.1111/nmo.70444)
Supplement: Supplementary file 1 [file NMO-38-e70444-s001.docx]

Supplementary Table 1. Setup parameters for cell detection in Chromogenic Immunohistochemistry

| Setup parameters  Detection Channel  Requested Pixel Size | Hematoxylin  0.2µm |
| --- | --- |
| Nucleus parameters  Background radius  Median filter radius  Sigma  Minimum area  Maximum area | 60µm  0  2.5  40µm^2  180µm^2 |
| Intensity parameters  Threshold  Split by shape | 10  Yes |
| Cell parameters  Cell expansion  Include cell nucleus | 5µm No |
| General parameters  Smooth boundaries  Make measurements | Yes  Yes |

Supplementary Table 2. Setup parameters for cell detection in Fluorescent Immunohistochemistry

| Setup parameters  Detection Channel  Requested Pixel Size | DAPI  0.2µm |
| --- | --- |
| Nucleus parameters  Background radius  Median filter radius  Sigma  Minimum area  Maximum area | 60µm  0  2.5  40µm^2  180µm^2 |
| Intensity parameters  Threshold  Split by shape | 5  Yes |
| Cell parameters  Cell expansion  Include cell nucleus | 5µm No |
| General parameters  Smooth boundaries  Make measurements | Yes  Yes |

Supplementary Table 3. Setup parameters for positive cell detection in Chromogenic Immunohistochemistry

| Setup parameters  Detection image  Requested Pixel Size | DAB  0.2µm |
| --- | --- |
| Nucleus parameters  Background radius  Median filter radius  Sigma  Minimum area  Maximum area | 60µm  0  2.5  40µm^2  180µm^2 |
| Intensity parameters  Threshold  Max background intensity  Split by shape | 0.1  2  Yes |
| Cell parameters  Cell expansion  Include cell nucleus | 5µm No |
| General parameters  Smooth boundaries  Make measurements | Yes  Yes |
| Intensity threshold parameters  Score Compartment  Threshold 1 | Cell: DAB OD Mean  0.5 (Adapted to background for each stain) |

Supplementary Table 4. Setup parameters for positive cell detection in Fluorescent Immunohistoechmistry.

| Object filter | Detections (all) |
| --- | --- |
| Channel filter | *Dependent on analyzed staining* |
| Measurement | Cell: *Staining* OD Mean |
| Threshold | *Calculated as stated in Materials and Methods section* |
| Above threshold | Positive |
| Below threshold | Negative |

Supplementary Table 5. Sum of analyzed HuC/D-positive neurons per patient and plexus

| **Patient** | **Sections analysed** | **MP neurons** | **ESP neurons** | **ISP neurons** | **Total neurons** |
| --- | --- | --- | --- | --- | --- |
| H1 | 2 | 158 | 52 | 94 | 304 |
| H2 | 2 | 437 | 147 | 157 | 741 |
| H4 | 2 | 370 | 36 | 114 | 520 |
| H5 | 2 | 269 | 237 | 205 | 711 |
| H6 | 2 | 299 | 20 | 29 | 348 |
| H7 | 1 | 224 | 82 | 78 | 384 |
| H8 | 2 | 90 | 48 | 16 | 154 |
| **Total** | **15** | **1847** | **622** | **693** | **3162** |

Supplementary Table 6. Comparison between Transitional and Ganglionic Zone Chromogenic Immunohistochemistry

| Plexus | Mean difference | Median difference | Wilcoxon adjusted p-value | T-test adjusted p-vale |
| --- | --- | --- | --- | --- |
| HuC/D Transitional vs. Ganglionic | | | | |
| PM | 13.8 | 6.96 | *0.0469* | 0.127 |
| PSE | 0.77 | 0.61 | 0.219 | 0.215 |
| PSI | 0.74 | 0.74 | 0.219 | 0.215 |
| nNOS Transitional vs. Ganglionic | | | | |
| PM | 9.8 | 7.7 | 0.891 | 0.614 |
| PSE | 0.13 | 0.27 | 0.891 | 0.838 |
| PSI | 0.8 | 0 | 0.891 | 0.636 |
| ChAT Transitional vs. Ganglionic | | | | |
| PM | 6.3 | 5.1 | 0.234 | 0.213 |
| PSE | 1.1 | 0.36 | 0.234 | 0.229 |
| PSI | 0.88 | 0.30 | 0.578 | 0.398 |

Supplementary Table 7. Children under 1 year, Comparison between Transitional and Ganglionic Zone Chromogenic Immunohistochemistry

| Plexus | Mean difference | Median difference | Wilcoxon adjusted p-value | T-test adjusted p-vale |
| --- | --- | --- | --- | --- |
| HuC/D Transitional vs. Ganglionic | | | | |
| PM | 20.2 | 15.4 | 0.375 | 0.183 |
| PSE | 1.4 | 1.6 | 0.375 | 0.110 |
| PSI | 0.69 | 0.60 | 0.375 | 0.294 |
| nNOS Transitional vs. Ganglionic | | | | |
| PM | 12.8 | 7.1 | 1 | 0.870 |
| PSE | 0.47 | 0.99 | 1 | 0.870 |
| PSI | 1.23 | 0.15 | 1 | 0.870 |
| ChAT Transitional vs. Ganglionic | | | | |
| PM | 7.4 | 6.1 | 0.5 | 0.675 |
| PSE | 1.3 | 0.56 | 0.375 | 0.675 |
| PSI | 0.86 | 0.62 | 0.875 | 0.675 |

Supplementary Table 8. Comparison between Transitional and Ganglionic Zone Fluorescent Immunohistochemistry

| Plexus | Mean difference | Median difference | Wilcoxon adjusted p-value | T-test adjusted p-vale |
| --- | --- | --- | --- | --- |
| HuC/D only Transitional vs. Ganglionic | | | | |
| PM | 2.7 | 1.2 | 0.0938 | 0.165 |
| PSE | 1.2 | 0.62 | 0.0938 | 0.165 |
| PSI | 0.80 | 0.87 | 0.0938 | 0.163 |
| HuC/D/nNOS Transitional vs. Ganglionic | | | | |
| PM | 0.65 | 0.73 | 0.75 | 0.774 |
| PSE | 0.04 | 0 | 1 | 0.883 |
| PSI | 0.25 | 0.08 | 0.141 | 0.249 |
| HuC/D/ChAT Transitional vs. Ganglionic | | | | |
| PM | 15 | 0.62 | 0.234 | 0.239 |
| PSE | 0.59 | 0.50 | 0.313 | 0.239 |
| PSI | 0.38 | 0.53 | 0.313 | 0.239 |
| HuC/D/nNOS/ChAT Transitional vs. Ganglionic | | | | |
| PM | 3.5 | 3.2 | 0.058 | 0.063 |
| PSE | 0.28 | 0.22 | 0.297 | 0.470 |
| PSI | 0.99 | 0.16 | 0.118 | 0.470 |

Supplementary Table 9. Children below 1 year, Comparison between Transitional and Ganglionic Zone Fluorescent Immunohistochemistry

| Plexus | Mean difference | Median difference | Wilcoxon adjusted p-value | T-test adjusted p-vale |
| --- | --- | --- | --- | --- |
| HuC/D only Transitional vs. Ganglionic | | | | |
| PM | 4.5 | 3.3 | 0.375 | 0.176 |
| PSE | 2.1 | 1.9 | 0.375 | 0.176 |
| PSI | 1.4 | 1.09 | 0.375 | 0.0961 |
| HuC/D/nNOS Transitional vs. Ganglionic | | | | |
| PM | 0.3 | 0.85 | 1 | 0.771 |
| PSE | 0.24 | 0.17 | 1 | 0.616 |
| PSI | 0.34 | 0.31 | 1 | 0.578 |
| HuC/D/ChAT Transitional vs. Ganglionic | | | | |
| PM | 2.1 | 1.7 | 0.375 | 0.352 |
| PSE | 0.92 | 1.1 | 0.5 | 0.352 |
| PSI | 0.61 | 0.59 | 0.5 | 0.352 |
| HuC/D/nNOS/ChAT Transitional vs. Ganglionic | | | | |
| PM | 2.9 | 2.8 | 0.375 | 0.255 |
| PSE | 0.44 | 0.29 | 0.375 | 0.584 |
| PSI | 1.7 | 0.5 | 0.375 | 0.584 |

Supplementary Table 10. Comparison of Proportions of each Neuronal Subtype between Transitional and Ganglionic Zone

| Zone | Mean difference | Median difference | Wilcoxon adjusted p-value | Paired t-test adjusted p-vale | Unpaired t-test adjusted p-value |
| --- | --- | --- | --- | --- | --- |
| HuC/D only Transitional vs. Ganglionic | | | | |  |
| PM | 8.5 | 0.8 | 1 | 0.697 | 0.512 |
| PSE | 4.3 | 9.7 | 1 | 0.709 | 0.747 |
| PSI | 10.7 | 7.5 | 0.0675 | 0.0641 | 0.269 |
| HuC/D/nNOS Transitional vs. Ganglionic | | | | |  |
| PM | 3.9 | 0.4 | 1 | 0.657 | 0.749 |
| PSE | 10.8 | 4.9 | 0.178 | 0.218 | 0.208 |
| PSI | 2.5 | 1.3 | 1 | 0.657 | 0.662 |
| HuC/D/ChAT Transitional vs. Ganglionic | | | | |  |
| PM | 4.4 | 2.2 | 0.615 | 1 | 0.450 |
| PSE | 2.4 | 2.9 | 1 | 1 | 0.510 |
| PSI | 11.0 | 3.2 | 1 | 1 | 0.913 |
| HuC/D/nNOS/ChAT Transitional vs. Ganglionic | | | | |  |
| PM | 8.0 | 6.7 | 0.894 | 0.668 | 0.514 |
| PSE | 1.4 | 0.2 | 1 | 0.726 | 0.835 |
| PSI | 7.1 | 5.8 | 0.108 | 0.145 | 0.469 |

Supplementary Table 11. Bayesian Modelling Estimation Parameters

|  | Estimate | Est. Error | lower-95% CI | upper-5% CI | Bulk_ESS | Tail_ESS |
| --- | --- | --- | --- | --- | --- | --- |
| muHuCDChAT_Intercept | -0.51 | 0.45 | -1.39 | -0.37 | 4052 | 4706 |
| muHuCDnNOS_Intercept | -0.31 | 0.60 | -1.48 | 0.89 | 3519 | 3791 |
| muHuCDnNOSChAT_Intercept | 0.58 | 0.52 | -0.43 | 1.61 | 3275 | 3457 |
| muHuCDChAT_plexusExternalSubmucosal | -0.39 | 0.45 | -1.28 | 0.50 | 5033 | 5758 |
| muHuCDChAT_plexusInternalSubmucosal | -0.20 | 0.43 | -1.04 | 0.65 | 4781 | 5649 |
| muHuCDChAT_zoneTransitional | -0.64 | 0.52 | -1.67 | 0.38 | 3625 | 4846 |
| muHuCDChAT_plexusExternalSubmucosal:zoneTransitional | 0.47 | 0.73 | -0.97 | 1.86 | 4050 | 5438 |
| muHuCDChAT_plexusInternalSubmucosal:zoneTransitional | 0.38 | 0.67 | -0.90 | 1.70 | 3793 | 5340 |
| muHuCDnNOS_plexusExternalSubmucosal | -1.34 | 0.50 | -2.35 | -0.37 | 5059 | 5888 |
| muHuCDnNOS_plexusInternalSubmucosal | -1.58 | 0.51 | -2.60 | -0.60 | 5265 | 6031 |
| muHuCDnNOS_zoneTransitional | -0.05 | 0.45 | -0.94 | 0.82 | 4233 | 5359 |
| muHuCDnNOS_plexusExternalSubmucosal:zoneTransitional | 1.05 | 0.70 | -0.30 | 2.45 | 4812 | 5856 |
| muHuCDnNOS_plexusInternalSubmucosal:zonetransitional | -0.46 | 0.74 | -1.91 | 0.95 | 4683 | 5886 |
| muHuCDnNOSChAT_layerExternalSubmucosal | -1.42 | 0.42 | -2.27 | -0.60 | 5386 | 6251 |
| muHuCDnNOSChAT_layerInternalSubmucosal | -1.67 | 0.43 | -2.54 | -0.85 | 5333 | 5370 |
| muHuCDnNOSChAT_zoneTransitional | -0.48 | 0.40 | -1.27 | 0.29 | 3930 | 5842 |
| muHuCDnNOSChAT_layerExternalSubmucosal:zoneTransitional | 0.69 | 0.64 | -0.57 | 1.94 | 4856 | 5926 |
| muHuCDnNOSChAT_layerInternalSubmucosal:zoneTransitional | 0.07 | 0.64 | -1.17 | 1.34 | 4811 | 5595 |

Supplementary Figure 1 Exemplary detailed depiction of automated neuronal subtype classification in a single ganglion in the myenteric plexus. Stainings shown are (a) DAPI, (b) DAPI/HuC/D, (c) DAPI/nNOS, (d) DAPI/ChAT. A combined image of all markers was used for classification (e), depicted are examples of all identified subtypes of HuC/D positive cells and nNOS positive cells negative for HuC/D.

Supplementary Figure 2. Spaghetti plot depicting the paired comparison of neuronal density (cells per mm) the neuronal subtypes investigated between ganglionic and transitional zone and each plexus in all patients: HuC/D^+^/nNOS^-^/ChAT^-^ (a), HuC/D^+^/nNOS^+^/ChAT^-^ (b), HuC/D^+^/nNOS^-^/ChAT^+^ (c), HuC/D^+^/nNOS^+^/ChAT^+^ (d) - overall median and IQR are shown in black. Analysis is split by plexus (MP = myenteric plexus, ESP = external submucosal plexus, ISP = internal submucosal plexus).
